# Getting Beyond Pros and Cons: Results of a Stakeholder Needs Assessment on Physician Assisted Dying in the Hospital Setting

**DOI:** 10.1007/s10730-022-09492-w

**Published:** 2022-08-23

**Authors:** Andrea Frolic, Leslie Murray, Marilyn Swinton, Paul Miller

**Affiliations:** 1 Program for Ethics and Care Ecologies (PEaCE), Hamilton Health Sciences, McMaster University Medical Center, 1F9-1200 Main Street West, Hamilton, ON L8N 3Z5 Canada; 2 Department of Health Research Methods, Evidence and Impact, McMaster University, Hamilton, ON Canada; 3 Mohawk College, Hamilton, ON Canada; 4 Medical Assistance in Dying Program, Hamilton Health Sciences, Hamilton, ON Canada; 5 Division of Emergency Medicine, McMaster University, Hamilton, ON Canada; 6 Department of Family Medicine, McMaster University, Hamilton, ON Canada

**Keywords:** MAiD, Medical Assistance in Dying, Physician Assisted Dying, Euthanasia, Hospitals, Stakeholders, Needs assessment, Mixed methods, Organizational ethics, Consultation

## Abstract

This study assessed the attitudes and needs of physicians and health professional staff at a tertiary care hospital in Canada regarding the introduction of physician assisted dying (PAD) during 2015–16. This research aimed to develop an understanding of the wishes, concerns and hopes of stakeholders related to handling requests for PAD; to determine what supports/structures/resources health care professionals (HCP) require in order to ensure high quality and compassionate care for patients requesting PAD, and a supportive environment for all healthcare providers across the moral spectrum. This study constituted a mixed methods design with a qualitative descriptive approach for the study’s qualitative component. A total of 303 HCPs working in a tertiary care hospital completed an online survey and 64 HCPs working in hospital units with high mortality rates participated in 8 focus group discussions. Both focus group and survey data coalesced around several themes to support the implementation of PAD following the decriminalization of this practice: the importance of high quality care; honoring moral diversity; supporting values (such as autonomy, privacy, beneficence); and developing resources, including collaboration with palliative care, education, policies and a specialized team. This study provided the foundational evidence to support the development of the PAD program described in other papers in this collection, and can be a model for gathering evidence from stakeholders to inform the implementation of PAD in any healthcare organization.

**Supplementary Information:**

The online version contains supplementary material available at 10.1007/s10730-022-09492-w.

## Introduction

On February 6, 2015 the Supreme Court of Canada ruled to decriminalize Physician Assisted Dying (PAD) in Canada. This decision was suspended for a year to allow Parliament to develop legislation to govern this new practice; this suspension was further extended by the court such that the new law governing PAD, or Medical Assistance in Dying (MAiD) as it was called later in Bill C-14, did not come into effect until June 2016 (Downie, 2022).^1^ Unfortunately, about 60% of Canadians die in hospitals (Statistics Canada, 2020), thus hospitals were forced to grapple with the question of how to deliver assisted dying services when PAD became decriminalized. Polls revealed that the majority of Canadians supported PAD, but most physicians did not (Attaran & Phil, 2015). This gap revealed a major risk for hospitals: patients were likely to request PAD once it became legally available, but it was unclear if any physicians would volunteer to provide the service. And if physicians did step forward, how would they be protected from the legal, moral, and emotional risks inherent in this new practice, especially in the context of a medical culture that remained largely opposed to it? It was also recognized that in the hospital setting, PAD could not be carried out by a physician alone, but would impact everyone in that patient’s circle of care: nurses, administrators, healthcare aides, all health professional staff and learners. Given the polarization around PAD, many were fearful of the impacts it could have on relationships within teams, and most importantly, on the end of life experiences of patients and families.

At Hamilton Health Sciences (HHS), a tertiary care hospital in Hamilton, Ontario, the Office of Clinical & Organizational Ethics was tasked with preparing the way for PAD organizationally, in collaboration with the Medical Advisory Committee (Frolic & Miller, 2022). HHS chose to approach the moral and social challenge of preparing for patient requests for PAD by designing an organizational ethics engagement process based in the values of the hospital and grassroots stakeholder engagement (Frolic & Miller, 2022). This engagement process included conducting a comprehensive, mixed methods needs assessment study with hospital staff, leaders and physicians. This stakeholder engagement process was called the “Physician Assisted Dying Readiness Assessment Project” (PADRAP). Its goal was to inform the design of PAD resources and structures based on an understanding of stakeholders’ fears, concerns, assumptions and needs in the interval between the Supreme Court decision and the initiation of the practice of MAiD. (Note: at the time of the study, no decision had been made regarding whether HHS would provide PAD; the hospital’s executive leadership team suspended that decision until the results of the PADRAP became available in February 2016.)

When this study was conducted, some published literature described the attitudes toward PAD of discrete healthcare professional (HCP) groups (see Curry, Schwartz,Gruman et al., 2000; Denier, Dierckx de Casterle, De Bal et al., 2009). However, in 2015, it was not apparent that any study had examined this issue in the context of the hospital setting, or attempted to collect, compare and contrast the attitudes of different HCPs working in hospitals. In addition, no study had been conducted with the express purpose of using stakeholder perspectives to inform the development of infrastructure to support clinicians to navigate the complex moral terrain of a new practice like PAD. In addition to filling an important gap in the literature on the practical ethics involved in the implementation of a contentious practice like PAD in an institutional setting, we hope that this needs assessment process could serve as a model for stakeholder engagement for any complex change in a health care system that requires an ethically-informed, reflective, and evidence-based approach to designing a new clinical practice.

### Study Objective and Goals

The goals of our mixed methods study (PADRAP) were to: (i) assess HCP readiness for PAD; (ii) identify their values, concerns, aspirations and needs surrounding this practice change; and (iii) use the study results to inform the development of structures (such as policies and resources) to help HCPs respond to patient requests for PAD. This study was part of a wider process of organizational ethics engagement designed to inform the creation of a high-quality, sustainable and accountable approach to MAiD care in the hospital context (Frolic & Miller, 2022).

Note that the purpose of the study was *not* to determine how many HCPs were “in favor” or “against” PAD, or to attempt to recruit PAD providers. The research team recognized that whether one is “in favor” or “against” anything is rarely absolute and may be determined by the specifics of the clinical situation, and the resources available to support a range of choices (Oliphant & Frolic, 2020). In addition, the purpose of the study was to identify common values held by all HCPs, and to develop infrastructure and resources to empower all stakeholders in navigating requests for PAD, regardless of their personal views on the topic. In this way, PADRAP aimed to defuse polarization by highlighting commonalities rather than differences and by focusing on the practical ethics of implementation rather than on philosophical pro/con positions.

## Methods

This Research Ethics Board approved study was conducted at an academic hospital comprised of six specialized healthcare facilities with 1145 beds and 13,000 staff and physicians. Approximately 2000 patients die every year at HHS, which is the largest tertiary care center for the Hamilton-Niagara region of Ontario and includes the largest palliative care unit in Canada. It has a strong emphasis on teaching and research, as well as a regional cancer program, large outpatient neurology, cardiac and geriatric programs, and a large acute medicine program.

This mixed methods study was overseen by the Physician Assisted Dying Working Group, comprised of physician leaders from key clinical programs (oncology, critical care, palliative care, medicine), pharmacy, nursing and inter-professional practice, as well as risk management and ethics. The role of the Working Group was to support recruitment to the study and to make recommendations to hospital senior leaders based on the results. The PADRAP included an anonymous online survey and qualitative focus groups. The anonymous, online survey was distributed by email through the hospital public relations department, and through clinical and physician leaders; it was open to all HCPs at HHS who provided care for adult patients from November 5th to December 18th 2015 (approximately 3000 people in total). The survey included questions about demographics and questions to elicit input about needs/values surrounding the possible implementation of PAD at HHS. (See Online Appendix 2 for the full survey tool.)

Focus group participants were purposively recruited (Patton, 2005) from adult clinical programs with the highest mortality rates in the hospital where requests for PAD were most likely to arise: oncology, internal medicine, palliative care, critical care and family medicine. Recognizing that physicians play a lead role in PAD and other health professionals provide crucial supportive care for patients, separate focus groups were held with physicians and other interprofessional staff working in these programs. Participants were never asked to disclose their personal views on PAD. The survey and focus groups included the same 5 open-ended questions. Quantitative data were tabulated anonymously using the Survey Monkey software. Open-ended survey data and focus group transcripts were analyzed using conventional qualitative content analysis (Hseih & Shannon, 2005) where codes are directly derived from the data rather than using preconceived categories. For more details about the methodology please refer to Online Appendix 1.

## Results

### Survey Participants

The online PADRAP survey was completed by 303 health care professionals, approximately one-third of respondents were physicians (35%), approximately one-third were nurses (33%) and approximately one-third were other health care professionals and staff (Table 1).

**Table 1:** Survey Participants

Profession	N = 303 # (%)
Physicians	107 (35.3)
Nurses	101 (33.3)
Other*	46 (15)
Social worker	16 (5.3)
Pharmacist	8 (2.6)
Clinical manager	7 (2.3)
Respiratory therapist	6 (2)
Occupational therapist	5 (1.7)
Physiotherapist	4 (1.3)
Spiritual care provider	3 (1)

^*^Other category included anyone who was in a profession that wasn’t listed and included administrative staff, laboratory staff, radiation therapists.

### Focus Group Participants

A total of 64 participants attended eight focus groups that were conducted over six weeks from November 5 to December 18, 2015. Six of the focus groups were comprised of homogeneous groups; five physician-only groups and a pharmacist-only group. This was done because physicians and pharmacists have specific, defined roles/responsibilities for PAD, and we wanted to provide a safe environment for participants to express their role-specific opinions and concerns. The other two focus groups were comprised of a mix of interprofessional staff working in family medicine, oncology, critical care, internal medicine and palliative care. These staff included: nurses, spiritual care providers, speech language pathologists, respiratory therapists, occupational and physical therapists, social workers and psychologists (see Table 2 for details). All focus group participants provided written consent.

**Table 2:** Focus Group Participants

	Participants *N* = 64
Critical care physicians	10
Oncology physicians	7
Medicine physicians	6
Palliative care physicians	5
Family physicians	2
Interprofessional healthcare providers	24
Pharmacists	10

## Findings

Although participants were never explicitly asked, a significant majority (> 90%) of survey participants spontaneously expressed general support for access to PAD for their patients in the open-ended questions, while also identifying the challenges that PAD posed in the hospital setting. Though participants self-selected for the study, it is felt that this data is significant as those with strong opinions on either end of the moral spectrum likely would be motivated to self-select to participate in the survey. Whatever their personal beliefs, participating HCPs shared similar values, hopes and needs regarding responding to patient requests for PAD across professions and medical specialties. Participants identified that the development of a specialized interprofessional PAD team is essential in the hospital context to ensure safe, high-quality care for patients and families, and to support the diverse values of the workforce by enabling referrals.

Both focus group and survey data coalesced around the following three themes: (i) health care professionals’ attitudes towards PAD, (ii) values informing PAD provision, and (iii) infrastructure and resources required to provide high quality PAD care.

The codes that inform these themes are summarized in Table 3.

**Table 3:** Codes and Themes from the PADRAP Qualitative Data Analysis

**Health care professionals’ attitudes towards PAD** • spectrum of opinion • respect for moral diversity amongst HCPs
**Values informing PAD provision** • respect for patient autonomy • privacy and confidentiality (for patients and PAD providers) • reduce suffering for patients and families (beneficence)
**Resources and infrastructure required to provide high quality PAD care** • access to palliative care and adequate symptom management • supports for patient decision-making • supports for families • supports for staff/physicians—creating a culture of respect for all • education on PAD for HCP and public • PAD policies/procedures • specialized PAD Consultation /Assessment Team

### Health Care Professionals’ Diverse Attitudes Towards PAD

Health care professional’s attitudes towards PAD varied across a wide spectrum of opinion. Although participants were never asked to disclose their personal views on PAD, most survey participants volunteered their opinions in the open-ended questions, likely because they felt safe doing so given the anonymity of the survey. A minority of survey participants indicated they were opposed to PAD under any circumstances. Most opposition was based on the belief that the preeminent role of healthcare professionals is to preserve life, as well as referencing personal religious/moral beliefs. A few illustrative comments include:

"It is against everything we learn as nurses."

"Our practices as physicians, our oath when we entered this profession, and even our name 'Health Sciences' are at odds with this movement of physician assisted dying."

"I believe God gives life and it is not up to us to take it."

Very few focus group participants expressed strong personal views about PAD, either for or against. The exception was in the palliative care physician focus group, where the majority of participants articulated objection to PAD under any circumstances, largely due to their concern that PAD undermines the values of palliative care, including finding meaning in the process of dying, and the privilege of caring for patients through a natural death. One palliative care physician described:

"In our society broadly, including in our healthcare system, we have a great deal of difficulty choosing to acknowledge that there can be value in suffering and to make a choice as individual practitioners and as a system is that sometimes our job is to bear witness to that suffering… How do we not swing in the sense of devaluing that…the people who choose that path and how do we continue to build our team, our health care providers across the organization…[to] continue to build their ability to be in the presence of suffering … [when] there is this other path that seems so easy, and right there and potentially seductive in a way, and may be the right thing to do in some situations…There is a potential risk I think that we could erode our ability to as practitioners and as an organization, to be present with people who are suffering."

The majority of focus group and survey participants of all professional backgrounds and specialties stated their support in principle for the hospital offering PAD to patients who meet legal criteria, *if the right safeguards were put into place*. All health profession groups identified several benefits of PAD including: honoring patient choice, facilitating earlier end of life conversations, and decreasing patient suffering. Some expressed unreserved support for PAD, indicating they were “*one hundred percent for the change*” and that they were “*delighted*” with the Supreme Court decision. Most, however, were more reserved in their support, stating that although they believed in access to PAD, they were unsure if they would be willing to participate directly in providing PAD. Many acknowledged that their decisions to provide or not provide PAD would be case-specific. One oncologist reflected:

"I am, worried about a patient wanting me to provide this and me not being, feeling I am able to do it. So, I have a patient right now actually I’m expecting will be asking me in February, and I think…for me I’m in a grey area. So, I think there is some I would be fine with it, and there is some I would not."

Regardless of their personal opinions, many focus group participants and survey respondents emphasized the need to ensure that the values of all staff are respected, including values around conscientious objection and participation in PAD. One internal medicine physician shared:

"I think it is important to have a position which allows people not to just adopt that “for or against” point of view, but to adopt a point of view which is, “I don’t wish to be involved”, with the provision that, as physicians, we need to ensure that patients’ needs get met."

A critical care physician expressed concern around “*the stigmatism [sic] of physicians that actually want to participate in this and feel that they want their practice to involve it, you know I worry about colleagues’ perceptions of people that want to help with this process.”* Participants suggested that education, along with specific policies/procedures should be developed to facilitate respect for the moral diversity of values and beliefs related to PAD, such as a conscientious objection guideline for all HCPs.

### Values Informing PAD Provision in the Hospital Context

Three values to inform PAD practice in the hospital context were identified from the survey and focus group data: (i) respect for patient autonomy, (ii) privacy and confidentiality for patients and PAD providers, and (iii) beneficence and reducing suffering.

#### Respect for Patient Autonomy

Clinicians in all focus groups spoke to how PAD respects patient autonomy. An internal medicine physician described one of the strengths of PAD as: *“it is the ultimate exercise of your autonomy that if you choose at some point to say ‘enough’, just as we would if someone chose to say enough chemotherapy or enough surgery…we would say okay fair enough, your choice*.”

This was echoed in a comment from a psychologist*: “I think I see the good that could come out of it for me is the sort of inherent level of respect for a human being’s wishes and desires which resonates really strongly with me.”*

#### Privacy and Confidentiality for Patients and PAD Providers

Participants from many of the focus groups identified the need for privacy and confidentiality in the implementation of PAD for everyone involved (patients and providers), and discussed challenges related to safeguarding privacy in an institutional setting.

Health care professionals in several focus groups spoke about the importance of patient privacy and believed that a patient’s plans for PAD should be kept confidential. However, it was recognized that this is a big challenge in a hospital setting. One of the pharmacists reflected:

"I keep thinking about the confidentiality piece with this because right now we have patients that share a bedroom…they can be in a very palliative state and they’re in a room with other people and their physicians are coming in and discussions are happening…it needs to be confidential for the person who is wanting to make that decision to go ahead with physician assisted dying but also for the other patients in the room who may be being cared for by these health care professionals providing this service - what are their thoughts with respect to that… ‘Oh my doctor is going to help this person die,’ they may not be comfortable with that."

Several HCPs suggested that PAD conversations take place in private areas and that PAD provision only take place in private rooms in the hospital setting to maintain confidentiality.

One of the critical care physicians emphasized a need for anonymity of PAD providers because of the potential for controversy:

"Our life has changed so much with internet and Facebook and all of the social media that didn’t exist when we marched down the abortion road and I think that we’re still going to have to be very careful about anonymity… we need to be careful that we’re not putting ourselves in jeopardy."

On the flipside, it was noted that it is important for some physicians to come forward to identify themselves as willing PAD providers, so that those who won’t provide PAD can provide referrals for their patients.

#### Beneficence and Reducing Suffering for Patients and Families

Many health professionals shared concerns about the suffering they witness in patients and their families at the end of life, and the potential for PAD to reduce that suffering. One occupational therapist described, “*Those patients are not dying imminently but we know they’re going to actually die, we know that they’re suffering, but it allows the conversation to open the door to those individuals*.” In addition, a nurse reflected how PAD could decrease suffering for families by allowing a patient to take on the responsibility for planning the end of their life, rather than leaving it up to families to make hard decisions, “*I think that the good of this is also that you take the burden of the decision making [away from families], the responsibility who live with the guilt of it. So hopefully you’ll see less suffering*.”

### Resources Required to Provide High Quality PAD Care in Hospital

Many participants identified a leadership role for hospitals in providing patients with access to PAD, as well as in developing resources to support PAD in the community. This leadership role was attributed to several features of tertiary care organizations, specifically: expertise in end-of-life care provision; their research and academic roles; access to interdisciplinary and specialty consultation; and their capacity to develop enabling infrastructure, such as policies, protocols, quality assurance systems and educational resources that can be shared with smaller organizations and community providers.

A number of resources to enable the provision of high-quality PAD in the hospital context were identified through focus group discussions. These included: access to palliative care, supports for patient decision-making, supports for families, supports for staff/physicians, education, policies and procedures to support PAD and having a specialized PAD consultation and assessment team.

#### Access to Palliative Care

Participants in every focus group articulated a need to develop more palliative care resources and related supports, both within the hospital and in the community, to help patients to get relief from their suffering, to ensure patients have legitimate options and to support them to make authentic end of life care choices. This call for better palliative care was also voiced frequently in the survey data.

Many focus group participants expressed concern about PAD distracting from developing a larger role for palliative care in the hospital. As one oncologist described:

"I’m concerned that there is so much focus and there is going to be so much media attention and everything else on this one group of patients which I think in other jurisdictions is such a small percentage of patients that we’re going to lose the bigger role of palliative care … we don’t have a national palliative care or even a provincial palliative care [strategy] that is accessible to our patients and that’s huge.…you’re going to lose that focus and that energy and time which there is only a certain amount of resources to put into this kind of thing."

Another participant, an occupational therapist, shared, “*My worry is that we don’t do palliative care well here already…the basics aren’t there yet so how can we add something else on?”.*

Participants in the palliative care focus group articulated concern that other parts of the hospital may assume they will be responsible for providing PAD (an assumption confirmed in our data), when in fact, many palliative care physicians are conscientious objectors to PAD (Eggertson, 2015). Palliative care physicians also feared that PAD may lead to incorrect perceptions about palliative care, as one participant described:

"I wonder if it’s going to change how we are going to treat the patients who are already palliative now, if it’s actually going to make palliation more difficult…. Now that this is an option it’s going to change the palliative perception that once palliative comes, they’ve come to give you a magic syringe to kill you."

Several respondents articulated a need to ensure that patients who are considering PAD have been fully educated on the natural course of their illness and informed about all the options available to them, including palliative care.

#### Education and Supports

Many focus group and survey participants underlined the need for ongoing education on a variety of topics related to PAD. Specific topics included: technical/clinical aspects, responding to requests for PAD, strategies for honouring the moral conscience of staff/physicians, and palliative care education for both clinicians and patients.

One of the oncologists reflected on how education has a role in creating a culture of respect for moral diversity:

"Having the space to be able to express opinions is important and how that happens I am not too sure at a corporate level but I mean it’s probably better for the needle to be on the over doing it side than the under doing it side in terms of opportunities like this to learn about and discuss the issue in here, the different views that are out there...the education and communication piece for this issue is going to be extremely important in order to develop the culture."

In addition, a large number of supports needed for PAD provision in the hospital setting were identified through this study; these were organized into three categories: supports for decision-making, family supports, and supports for staff and physicians. These are summarized in Table 4.

**Table 4:** Supports Needed for PAD Provision in the Hospital

Group	Supports needed
Patient Supports	• assistance with navigating end of life care options
	• access to psychiatry, social workers or psychologists to: assess/treat underlying depression or anxiety; assess capacity; and address existential suffering and anticipatory grieving
	• access to communication aids, speech pathologist, and translators to ensure their voice is heard
	• supports to fulfill final wishes before death, such as visits with family, final meals, etc
	• spiritual support to help patients to prepare for death and perform rituals and cultural practices
	• support for those found not eligible for PAD, but who perceive themselves as suffering intolerably
	• supports to ensure continuity of care, so patients don’t feel abandoned whatever the outcome of their PAD request
	• ethics consultation service for controversial or ambiguous PAD cases
Family Supports	• education about the legal requirements and process of PAD
	• emotional and psychological support for family members at all stages of the process
	• resources to enable families to assist patients to make choices that align with their goals and wishes
	• assistance with grieving/bereavement
	• assistance when there is conflict within the family about a patient’s PAD decision
	• education/reassurance for other patients and families in the patient’s room/ward
Staff and Physician Supports	• need for ongoing dialogue and reflection on the topic
	• supports to address concerns about the complexity of assessing patient competency in the context of PAD
	• staff and public education about the option of PAD
	• the need to create a non-judgemental culture of respect for all moral views

#### Policies and Procedures to Support PAD

Many respondents expressed the need for clear, transparent and accessible procedures and policies to ensure a high-quality and safe experience for patients seeking PAD and their families. A number of participants identified concerns about the “slippery slope”, and fear that without proper safeguards for patient autonomy and informed consent, vulnerable patients could be at risk of coercion or exploitation.

Participants also stated that infrastructure is needed to support high-quality PAD care, including protocols that ensure processes are clearly documented, standardized and evaluated, to ensure they are followed consistently and rigorously. One survey response summarized the infrastructure needed:

“Process identified; checklist/form/formal process essential to maintain public trust and confidence, as well as allowing a high standard of care that could easily be tracked and documented in a meaningful inter-professional way.”

At the same time, some respondents recognized a need to balance the rigor of the process with providing timely access to PAD, given these patients are suffering intolerably.

Participants in our readiness assessment suggested a number of processes to ensure high quality PAD provision in the hospital setting, these are summarized in Table 5.

**Table 5:** Specific Processes to Ensure High Quality PAD Provision in Hospital

• clear and timely referral processes for physicians/staff
• option of patient self-referral
• rigorous assessment processes for patient eligibility for PAD based on legal requirements, including psychological assessment in select cases where mental health is a concern
• thorough patient capacity assessment
• standards to ensure informed consent
• exploration of all treatment options
• documentation standards for the whole PAD process

#### PAD Consultation and Assessment Team

The need for a specialized team to support the provision of PAD in the hospital emerged through both the open-ended survey data and the focus group discussions. There was unanimity amongst respondents that patient requests for PAD should be supported by a specialized interdisciplinary team, with the right skills, knowledge and resources to support the patient, family, and other healthcare providers in the circle of care. Many participants expressed worry that they or their colleagues could be forced to perform or assist in PAD against their moral and religious beliefs. Referral to a specialized team of volunteers would protect conscientious objection, and minimize conflict between “willing” and “unwilling” staff/physicians within the patient’s circle of care. This team would have specific responsibilities for assessing the patient’s eligibility and performing PAD.

A number of different attributes for this specialized PAD consultation and assessment team were identified in the readiness assessment, these are summarized in Table 6.

**Table 6:** Attributes for the PAD Consultation and Assessment Team

• referrals accepted from physicians and from patients directly (self-referral)
• all PAD team members are willing providers
• diverse inter-professional membership (physicians, nurses, social workers, pharmacists, psychologists, ethicists, chaplains, etc.) to support whole person care of patient
• involvement of others in the patient’s circle of care, as appropriate (specialists and family physicians, especially if the patient wishes to die at home)
• institutional resources provided (i.e., funding, protected time) to ensure quality and sustainability of service

Participants also identified that the PAD team itself would need specific supports, including: appropriate education to ensure  competence, safety/security/confidentiality, psychological support, peer support, flexibility in workload/scheduling to accommodate PAD-related work, access to legal advice and support from an ethicist for challenging cases.

## Discussion

**Fig. 1:**
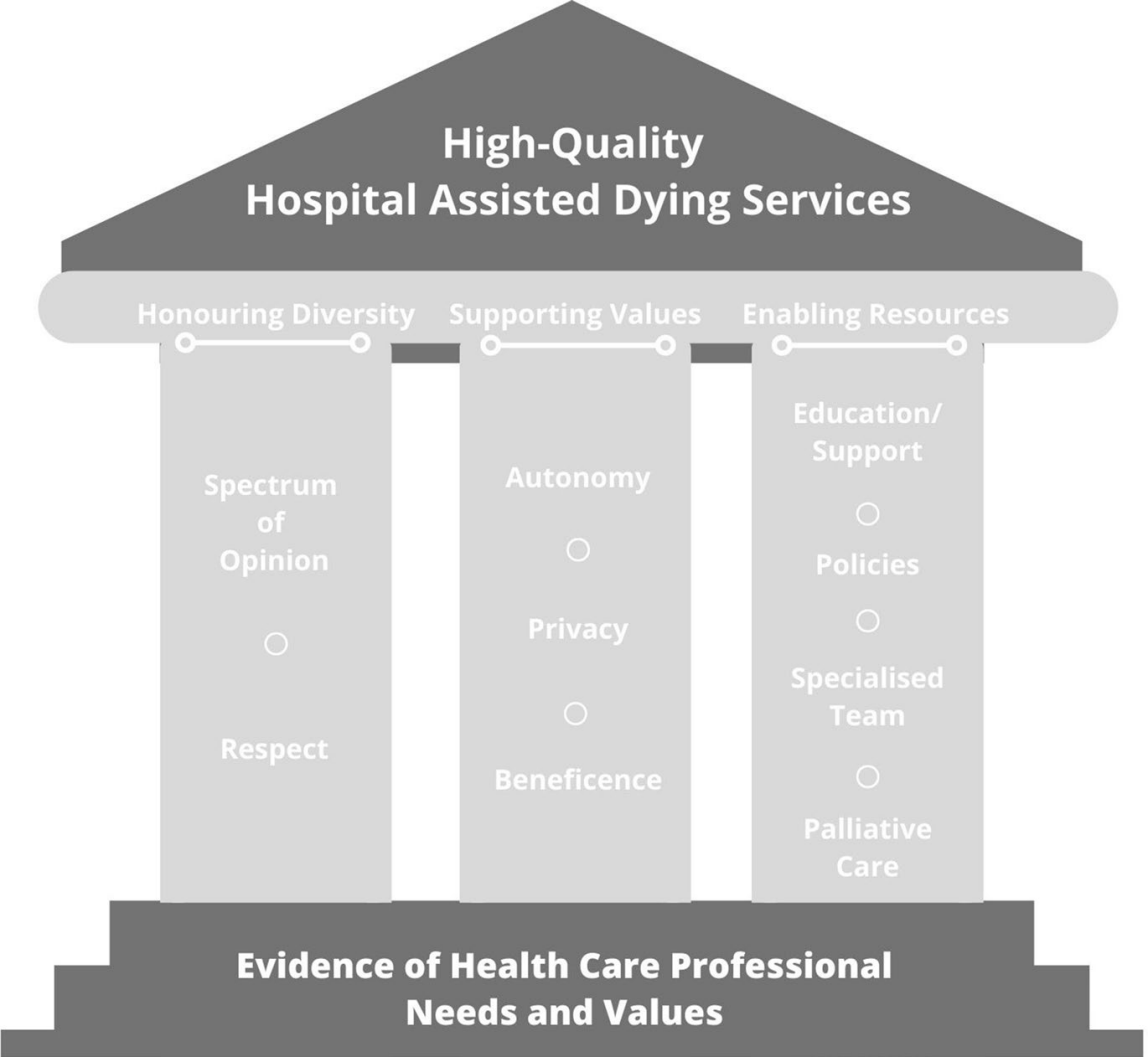
Designing Assisted Dying Services for Hospitals

The Physician Assisted Dying Readiness Assessment Project was an innovative study that was successful in engaging HCPs in reflecting on the potential impact of PAD on their practice, identifying common values, needs and concerns across the moral spectrum of opinions. The study enabled stakeholders to express a wide diversity of attitudes, and to find common ground in identifying three pillars to support high quality assisted dying services for the hospital setting. These pillars are: Honoring Diversity, Supporting Values and Enabling Resources, as summarized in Fig. 1.

Results of the PADRAP were presented to the Working Group, who drafted recommendations for the development of infrastructure and resources to support access to high-quality PAD, which were eventually approved by the hospital’s Medical Advisory Committee, executive leadership team and board of directors. These recommendations were put into place through the development of the Assisted Dying Resource and Assessment Service in 2016. Five years after its inception, these values continue to inform the delivery of high-quality assisted dying services at HHS (Frolic et al., 2022; Frolic et al., 2022a, b).

One of the strengths of our research is how we purposively sampled our focus group participants (Patton, 2005) from five medical programs that we anticipated would have the most involvement with PAD in the hospital setting (palliative care, internal medicine, critical care, oncology, family medicine) as well as pharmacy.

Conducting open-ended focus group discussions and collecting survey data allowed for triangulation of quantitative and qualitative data to assess the readiness of HCPs across a large organization, to identify common values and needs, and to identify practical strategies and steps to prepare staff/physicians for this controversial practice change. Triangulation amidst this data set supported thematic reliability (Tracy, 2010).

Another strength of our research is the open-ended, exploratory method we used to engage diverse healthcare professionals. Our research had no agenda beyond giving voice to staff and physician views on the possible provision of PAD in the hospital. While some emerging literature addresses clinician attitudes towards euthanasia provision (van Marwick et. al, 2017; Lowes, 2016; Oliphant & Frolic, 2020) this paper offers a novel contribution to the literature by proposing a method for exploring specific needs and supports to facilitate the design and sustainability of a hospital-based PAD program. Through this study we discovered HCPs are very willing to engage in these challenging conversations when they are carefully facilitated to ensure safety and respect and when they are invited to offer pragmatic solutions to inform the quality of PAD services, as well as identifying potential barriers and challenges. This needs assessment process set a tone for the culture of practice of PAD at HHS, by inviting deep listening and reflection, by focusing on common values rather than polarized opinions, and by identifying concrete resources and supports to enable the PAD in unique setting of a hospital, while also honoring the moral diversity of staff.

There were some limitations to this study. This study was conducted in a multi-site tertiary health care system located in a mid to large city in Canada and may not be transferable to community hospitals and rural sites that lack the resources and infrastructure to implement a needs assessment process. Furthermore, there was a limited number of family physicians involved in the study. Lastly, due to the time pressure of impending legislative changes at the time of this study, we could not involve more participants, such has clinical leaders, in the focus groups. Although, we did reach saturation in both the focus groups and survey data, there may have been additional perspectives that were not collected.

## Conclusions

In spite of the fact that the majority of Canadians die in hospital, there is a dearth of literature about the attitudes of hospital staff and physicians towards PAD. In addition, little is known about what HCPs working in institutional settings need in order to support patients requesting PAD, and to deliver high-quality PAD care. This study fills an important gap in the ethics literature by naming the fears, concerns, hopes and aspirations of HCPs regarding *how* PAD ought to be delivered. By focusing on the analysis of common values across the moral spectrum, and the identification of concrete supports to both promote access to PAD and preserve the conscience of clinicians, this study is an example of how practical ethics, stakeholder engagement and research methods can be combined to develop novel solutions to ethical problems in institutional care environments.

In any jurisdiction contemplating legalizing PAD, hospitals and other healthcare organizations, like hospices and long-term care facilities, play an important role in facilitating access to this end of life care option. Amendments to the Canadian law in 2021 (Government of Canada, 2021) expanded access to assisted dying to new populations (specifically patients who meet all eligibility criteria but who do not have a “reasonably foreseeable natural death”). Thus we anticipate that many healthcare facilities in Canada that have never entertained a request for assisted dying from a patient—such as rehabilitation hospitals, in-patient mental health facilities, addiction treatment centres, chronic pain clinics, residences for disabled persons, etc.—will be confronted with the same moral angst that our hospital grappled with in 2015–2016. This study provides a model for stakeholder engagement in the design of clinical services that are morally contentious, to deliver high-quality care without creating divisions within teams or exacerbating the polarization of organizational culture. PADRAP provides a method of grassroots engagement that we believe is replicable, transferable, and adaptable to any healthcare setting facing the prospect of responding to patient requests for physician assisted dying. It could also be adapted to assess the needs of stakeholders on any issue that is novel or potentially divisive, including the introduction of new genetic technologies, reproductive care options, or clinical services for historically stigmatized populations.

## Supplementary Information

Below is the link to the electronic supplementary material.

Supplementary file1 (DOCX 50 KB)

## Footnotes

1 Note on language: Throughout the paper we use the terms Medical Assistance in Dying (MAiD) (the term used in the 2016 Canadian federal legislation that governs MAiD practice [called Bill C-14 in 2016]) interchangeably with Physician Assisted Dying (PAD) (the term used in the 2015 Supreme Court Decision *Carter v Canada* that decriminalized assisted dying).
