# Supplementary material for: Getting Beyond Pros and Cons: Results of a Stakeholder Needs Assessment on Physician Assisted Dying in the Hospital Setting
Source: HEC Forum. 2022 Aug 23;34(4):391–408. doi: 10.1007/s10730-022-09492-w (PMC9671973; doi:10.1007/s10730-022-09492-w)
Supplement: Supplementary file 1 — Supplementary file1 (DOCX 50 KB) [file 10730_2022_9492_MOESM1_ESM.docx]

**Appendix 1**

**Detailed Methodology for the PADRAP Readiness Assessment**

*Design*

Mixed methods designs are most appropriate for understanding social data (such as behaviors, values, beliefs and attitudes) in complex environments and with heterogeneous populations. Physician Assisted Dying represents a complex social phenomenon as a new medical practice, one that impacts a wide variety of stakeholders; thus, survey methods alone cannot capture the complexity and variety of attitudes and values held by health professionals. A mixed method design allows for participants who wish to remain anonymous to share their perspectives, and generates more generalized information about attitudes, beliefs and needs across diverse groups. Focus groups yield thick description of stakeholder perspectives, and provide the basis for nuanced analysis and practical recommendations. The study employed qualitative description, a method which aims to present findings in the language of the participants with minimal theoretical interpretation (Sandelowski, 2000; Neergaard, Olesen, Andersen & Sondergaard, 2009).

*Sampling and Recruitment*

An email stating the purpose and importance of completing the online survey along with a consent statement and a link to the online survey was distributed through email lists to physicians and healthcare professionals working with adult patients at HHS during the study period (approximately 3000 people in total). Physicians and healthcare professionals working in pediatrics were not sent an invitation to participate in the survey because PAD was not decriminalized for persons under eighteen years of age. Clinical managers/educators and physician leaders supported recruitment by encouraging participation at staff meetings, posting recruitment posters and circulating reminder emails. It was also advertised institution wide in the weekly hospital news and on the homepage of the ethics department.

We used a combination of convenience and purposive sampling for the focus groups (Patton, 2002). Physicians and healthcare professionals were invited to participate in a focus group discussion by the PADRAP working group members and their practice chiefs through general departmental email lists.

**Focus Group Data Collection and Analysis**

The focus group questions were based on the study objectives and survey questions. The questions were reviewed for content validity by all co-investigators and pilot-tested with select physicians and health professionals. Written informed consent was obtained from all participants. Given the sensitivity of the conversation, the facilitator outlined ground rules to keep the conversation safe and respectful, and emphasized the confidentiality of the sessions, the anonymity of the data, and the option to decline to answer any questions. The length of the focus groups ranged from 90-120 minutes. The research coordinator took detailed field notes. All focus groups were digitally recorded, transcribed verbatim and anonymized for analysis (see Frolic & Miller [2022] for further information on focus group management)

Two investigators independently completed line-by-line coding of two transcripts and by consensus, developed the initial list of codes. The two investigators used this initial list of codes to code the remaining six transcripts (each investigator coded three of the six transcripts), meeting weekly to discuss new codes and insights and through consensus develop the final list of codes. All changes throughout the coding process were documented in an audit trail (Rodgers & Cowles, 1993) and new codes were applied to previously coded transcripts. All three investigators met bi-monthly to organize the codes into meaningful themes and discuss potential relationships between the codes – a process known as axial coding (Corbin & Strauss, 1990). N’Vivo Version 11.0 (QSR International, 2019) was used for data management and analysis. Data saturation was evaluated at the bi-monthly research team meetings through review of the transcripts and coding reports. All three investigators agreed that no new themes or insights were being identified in the data after the seventh focus group, indicating that data saturation had occurred, and the sample size was sufficient.

All three investigators reviewed coding reports from survey data analysis conducted by one investigator, and findings were discussed in the context of the focus group data, a process known as triangulation (Thurmond, 2001).

**Appendix 2**

**The Online Survey**

| 1. What is your profession?   \| clinical manager  nurse  occupation therapist  pharmacist  physician  physiotherapist  respiratory therapist  social worker  spiritual care provider  \| \| --- \| \| Other (please specify)  \|   2. How many years' experience do you have in your profession?   \| 1–5 years  6–15 years  16 plus years  \| \| --- \|   3. What most worries you about physician assisted dying, specifically, if it were practiced at HHS? (open text)  4. What do you see as the possible roles for Hamilton Health Sciences (HHS) in a system where physician assisted dying is an option for patients? (open text)  5. What supports would you want in place to ensure patients seeking physician assisted dying (PAD) receive safe/high quality care? (How might HHS interface with community supports for patients, including family physicians?) (open text)  6. What supports would you want in place for your clinical colleagues (physicians and other health professionals) who participate in physician assisted dying? (open text)  7. How can we cultivate a culture of respect for those colleagues who may participate in physician assisted dying and those who don’t (i.e. ensure respect for moral diversity)? (open text) |
| --- | --- | --- | --- |
